# A genistein-enriched diet neither improves skeletal muscle oxidative capacity nor prevents the transition towards advanced insulin resistance in ZDF rats

**DOI:** 10.1038/srep22854

**Published:** 2016-03-14

**Authors:** Bianca W. J. van Bree, Ellen Lenaers, Miranda Nabben, Jacco J. Briedé, Johanna A. Jörgensen, Gert Schaart, Patrick Schrauwen, Joris Hoeks, Matthijs K. C. Hesselink

**Affiliations:** 1 Department of Human Biology, NUTRIM School for Nutrition, Toxicology and Metabolism, Maastricht University, Maastricht, The Netherlands; 2 Department of Human Movement Sciences, NUTRIM School for Nutrition, Toxicology and Metabolism, Maastricht University, Maastricht, The Netherlands; 3 Department of Toxicogenomics, GROW School of Oncology and Developmental Biology, Maastricht University, Maastricht, The Netherlands

## Abstract

Genistein, a natural food compound mainly present in soybeans, is considered a potent antioxidant and to improve glucose homeostasis. However, its mechanism of action remains poorly understood. Here, we analyzed whether genistein could antagonize the progression of the hyperinsulinemic normoglycemic state (pre-diabetes) toward full-blown T2DM in Zucker Diabetic Fatty (ZDF) rats by decreasing mitochondrial oxidative stress and improving skeletal muscle oxidative capacity. Rats were assigned to three groups: (1) lean control (CNTL), (2) fa/fa CNTL, and (3) fa/fa genistein (GEN). GEN animals were subjected to a 0.02% (w/w) genistein-enriched diet for 8 weeks, whereas CNTL rats received a standard diet. We show that genistein did not affect the overall response to a glucose challenge in ZDF rats. In fact, genistein may exacerbate glucose intolerance as fasting glucose levels were significantly higher in fa/fa GEN (17.6 ± 0.7 mM) compared with fa/fa CNTL animals (14.9 ± 1.4 mM). Oxidative stress, established by electron spin resonance (ESR) spectroscopy, carbonylated protein content and UCP3 levels, remained unchanged upon dietary genistein supplementation. Furthermore, respirometry measurements revealed no effects of genistein on mitochondrial function. In conclusion, dietary genistein supplementation did not improve glucose homeostasis, alleviate oxidative stress, or augment skeletal muscle metabolism in ZDF rats.

A variety of natural dietary strategies have been examined to antagonize the progression of type 2 diabetes mellitus (T2DM)^1^,^2^. As such, in type 2 diabetic humans the consumption of soy has been reported to have beneficial effects as it lowers fasting insulin levels and HOMA-IR, a surrogate marker for insulin resistance^3^,^4^,^5^,^6^. However, which constituent of soy mediates these protective effects and its related mechanisms remains unclear^7^. There are indications that the most abundant and active phytoestrogen in soy, genistein^8^, could be beneficial in the management of T2DM by influencing skeletal muscle oxidative capacity. *In vitro* studies have shown that genistein can affect both glucose and lipid metabolism in skeletal muscle tissue^2^. Likewise, genistein stimulated glucose uptake in L6 myotubes independently of insulin under normoglycemic (5.5 mM) and hyperglycemic (25 mM) conditions in a dose-dependent manner^9^. Moreover, genistein was demonstrated to increase palmitate oxidation in C2C12 myotubes^10^. Animal models of T2DM also indicate that ingestion of a genistein-enriched diet could improve glucose homeostasis^11^,^12^,^13^,^14^ by reducing hyperglycemia, circulating insulin levels^12^,^15^,^16^, and hepatic lipid accumulation^17^,^18^. Additionally, genistein has been reported to have antioxidant activities, either *in vitro*^19^ or *in vivo* in liver and brain of insulin-resistant rodents^17^,^20^.

Although beneficial effects of genistein on glucose and fat homeostasis have been reported previously, the mechanisms underlying its beneficial effects remain poorly understood but include augmentation of mitochondrial oxidative capacity and ROS management. Interestingly, compromised mitochondrial function in skeletal muscle has been reported in models of insulin resistance and T2DM^21^,^22^ and may even precede overt T2DM. Since skeletal muscle glucose uptake is the central factor in maintenance of insulin sensitivity and development of T2DM, it will be important to elucidate the role of genistein in skeletal muscle mitochondrial capacity in a model of progressive insulin resistance. Therefore, the aim of this study was to investigate whether genistein influences glucose tolerance, and prevents or reduces the progression of the hyperinsulinemic normoglycemic state (pre-diabetes) towards full-blown T2DM by improving skeletal muscle oxidative capacity and alleviating oxidative stress. To this end, diabetes prone Zucker Diabetic Fatty (ZDF) rats were provided a genistein-enriched diet, starting at the age of 6 weeks, when rats were still normoglycemic. Our data show that consumption of a diet with 0.02% (w/w) genistein does not affect whole-body glucose tolerance, mitochondrial skeletal muscle oxidative capacity or -ROS production. Moreover, dietary genistein does not antagonize the pathogenesis of insulin resistance in ZDF rats.

## Results

### Animals

**Figure 1:**
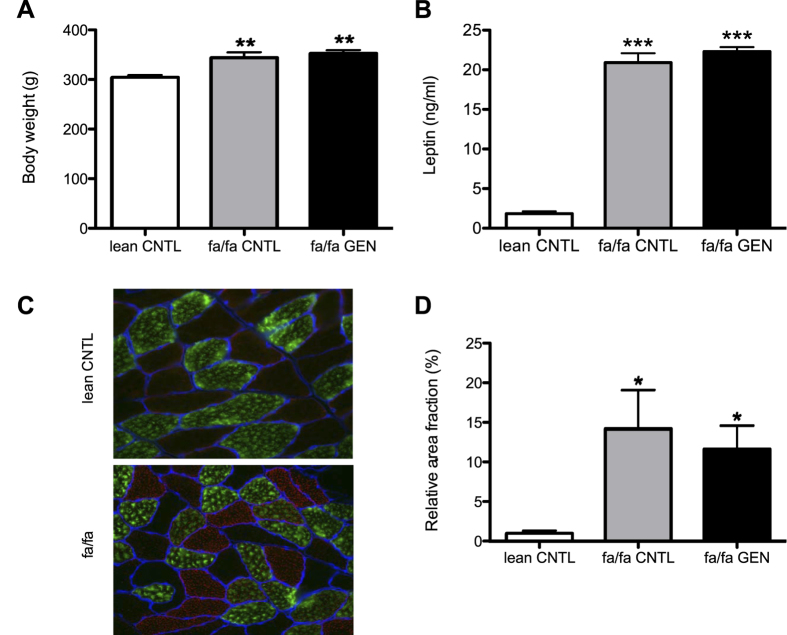
Dietary genistein consumption does not affect body weight or intramyocellular lipid content during maturation of ZDF rats. (**A**) Body weights and (**B**) plasma leptin levels of rats at the end of the 8-week dietary intervention. (**C**) Representative microscope images of ORO staining in gastrocnemius muscle sections from lean CNTL (upper panel) and fa/fa CNTL (lower panel) rats. (**D**) Quantification of IMCL content in gastrocnemius muscle expressed as percentage relative area fraction. The relative area fraction of IMCL content in lean CNTL rats was set to 1.0%. *P < 0.05 vs. lean CNTL, **P < 0.01 vs. lean CNTL, ***P < 0.0001 vs. lean CNTL.

Lean and fa/fa rats (aged 6 weeks) were placed on the standard Purina diet and body weight was monitored weekly throughout the 8-week intervention period. To study the effect of genistein on weight gain, a second group of fa/fa rats received the Purina diet supplemented with 0.02% (w/w) genistein. At 14 weeks of age, we observed that fa/fa rats consuming the genistein-enriched Purina diet had a similar body weight (353 ± 6g) in comparison to the fa/fa rats on the standard Purina diet (344 ± 11g) (Fig. 1A). Genistein addition to the diet did not influence food consumption in the fa/fa animals (data not shown). In comparison to the lean control (CNTL) animals, the fa/fa rats had a ~10% higher bodyweight (P = 0.001) (Fig. 1A) which can be attributed to the leptin receptor deficiency, resulting in a significantly higher food intake (39 g/day vs. 20 g/day respectively) despite high circulatory leptin levels in fa/fa animals (Fig. 1B, P < 0.0001).

Progression of insulin resistance during maturation of diabetes prone ZDF rats has previously been reported to occur in parallel with increased IMCL content^23^,^24^. Moreover, genistein has been suggested to promote lipid oxidation^25^, hence we also assessed the effect of genistein on skeletal muscle fat content (Fig. 1C). In line with previous observations we observed profoundly higher IMCL levels in fa/fa CNTL and fa/fa GEN rats than in lean CNTL rats (Fig. 1D, P < 0.05). Dietary supplementation of genistein, however, did not affect IMCL content significantly.

### Whole body glucose tolerance

The fa/fa ZDF rat is a recognized model of progressive insulin resistance during maturation^23^. At 6 weeks of age fa/fa rats are known to be hyperinsulinemic and normoglycemic, reflecting a pre-diabetic state, whereas at 14 weeks of age fa/fa rats suffer from severe hyperglycemia^24^. As cell studies indicate that genistein promotes glucose uptake in myocytes *in vitro*^9^, we studied the hypothesis that genistein would also promote glucose tolerance *in vivo*. Hence we tested if consumption of dietary genistein during maturation affects whole-body glucose tolerance by performing i.p. glucose tolerance tests (IPGTT) in rats at 13 weeks of age.

**Figure 2:**
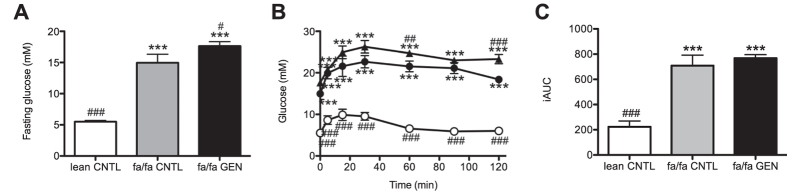
Dietary genistein consumption does not improve whole-body glucose tolerance in fa/fa rats at the age of 13 weeks. (**A**) 4-hr fasting glucose levels (mM) at T = 0 of the IPGTT after 7 weeks of dietary intervention. (**B**) Glucose tolerance curves of lean CNTL (open circles), fa/fa CNTL (closed circles) and fa/fa GEN (closed triangles) treated animals. (**C**) Analysis of the incremental area under the curve (iAUC) in groups as indicated. ***P < 0.0001 vs. lean CNTL, ^#^P < 0.05 vs. fa/fa CNTL ^##^P < 0.01 vs. fa/fa CNTL, ^###^P < 0.0001 vs. fa/fa CNTL.

As anticipated, fasting blood glucose levels were significantly higher in fa/fa rats compared to lean rats (16.4 ± 0.8 mM and 5.5 ± 0.2 mM respectively, P < 0.0001, Fig. 2A). Interestingly, and in contrast to what was anticipated based upon studies by others^9^,^13^,^15^, we observed that dietary supplementation of genistein resulted in significantly higher fasting blood glucose levels in genistein treated fa/fa rats (fa/fa GEN, 17.6 ± 0.7 mM) than in fa/fa rats fed the control diet (fa/fa CNTL, 14.9 ± 1.4 mM), P < 0.05, Fig. 2A).

Next we analyzed the blood glucose response to an intraperitoneal glucose challenge (IPGTT). An interaction effect was found between time and intervention on blood glucose levels (P < 0.0001, Fig. 2B), indicating differences in rise and clearance of plasma glucose between groups upon the i.p. glucose bolus. The glucose intolerant state of the fa/fa rats was confirmed by the significantly higher blood glucose concentration at all time points during the IPGTT in both fa/fa groups compared to the lean CNTL group (One-way ANOVAs p < 0.0001, Fig. 2B). Although the overall response (as is reflected by the iAUC) to an intraperitoneal glucose load was not affected by genistein, glucose levels at 60 (P = 0.002) and 120 minutes (P < 0.0001) after the glucose bolus were significantly higher in fa/fa GEN rats than in fa/fa CNTL rats (Fig. 2B). Despite these unfavorable effects of genistein on fasting glucose and glucose levels at t = 60 min and t = 120 min, the incremental area under the time-glucose curves (Fig. 2C) was not significantly different between fa/fa rats consuming the control diet and genistein-treated; 709 ± 8 and 765 ± 3 in fa/fa CNTL and fa/fa GEN rats respectively (Fig. 2C).

### Oxidative stress

**Figure 3:**
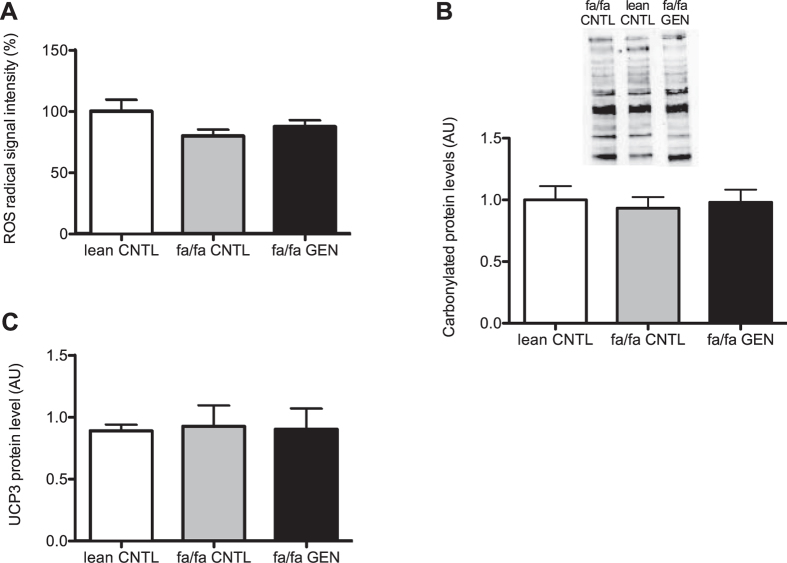
8 weeks of genistein feeding had no effects on skeletal muscle oxidative stress. (**A**) Superoxide anion radical production on malate with pyruvate as substrates was measured using ESR spectroscopy. The ROS radical signal intensity of the lean CNTL rats was set to 100%. (**B**) Quantification of oxidative stress in gastrocnemius muscles by analysis of carbonylated protein levels with Oxyblot. The carbonylated protein content in skeletal muscle of lean CNTL rats was set to 1.0 (AU). (**C**) Protein UCP3 expression levels in gastrocnemius muscle quantified by western blot analysis (AU).

Genistein has been reported to have antioxidant activity *in vitro* as well as *in vivo* in liver and brain tissues of insulin resistant rodents^17^,^19^,^20^. To explore whether genistein was able to also blunt mitochondrial ROS production in skeletal muscle, we measured superoxide anion radical production in a direct manner in isolated mitochondria from gastrocnemius muscles, at a high proton gradient (state 4 conditions, by ESR spectroscopy). This direct measure of superoxide production, however, revealed similar levels of skeletal muscle mitochondrial superoxide production in genistein-treated animals compared to animals on a control diet (Fig. 3A, P = 0.15). Thus, no differences were found between lean CNTL vs. fa/fa or between fa/fa CNTL and fa/fa GEN animals.

Next to mitochondrial ROS production, we also measured oxidative protein damage by quantifying carbonylated protein content through Oxyblot. In accordance with similar levels of mitochondrial superoxide generation, carbonylated protein content remained unaffected upon genistein-treatment (0.93 ± 0.09 vs. 0.98 ± 0.10 in fa/fa CNTL vs. fa/fa GEN rats respectively) (P = 0.89, Fig. 3B). Moreover, the fa/fa and lean CNTL had similar levels of carbonylated proteins. Jointly, these data suggest that genistein does not affect the antioxidant capacity in rat skeletal muscle.

### Mitochondrial uncoupling

UCP3 has been proposed to modulate lipotoxicity by alleviating proton gradient built-up^26^,^27^,^28^ thereby blunting the production of superoxide. This, along with the observation in C2C12 cells that genistein induces gene expression of UCP3 in C2C12 myotubes^10^, prompted us to measure UCP3 protein content. UCP3 protein content, however, was not affected by genistein (P = 0.98, Fig. 3C).

### Skeletal muscle mitochondrial density and function

**Figure 4:**
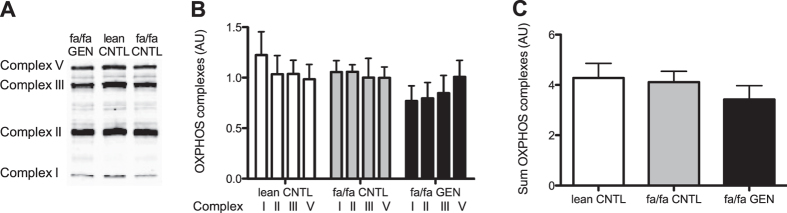
Similar mitochondrial densities in 14-week-old lean CNTL, fa/fa CNTL, and fa/fa GEN groups as were estimated by quantifying complexes I, II, III, and V of the mitochondrial respiratory chain (OXPHOS). (**A**) Representative western blot. (**B**) Quantification of western blot analysis of protein expression of OXPHOS complexes (AU) in gastrocnemius muscle homogenates and (**C**) sum of OXPHOS complexes (AU) in lean and fa/fa rats.

Subsequently, we analyzed the putative effect of genistein on skeletal muscle mitochondrial density and function. Mitochondrial density was estimated by quantifying protein levels of the structural components of the complexes involved in oxidative phosphorylation (OXPHOS) (Fig. 4A). Protein content of OXPHOS complexes was comparable in all groups (One-way ANOVAs, complex I P = 0.2, complex II P = 0.39, complex III P = 0.71, or complex V P = 0.99), regardless of the genotype (Fig. 4B). Also the sum of OXPHOS complexes was not different between groups (P = 0.49, Fig. 4C). Thus, the mitochondrial density seemed not significantly affected by genistein administration.

**Figure 5:**
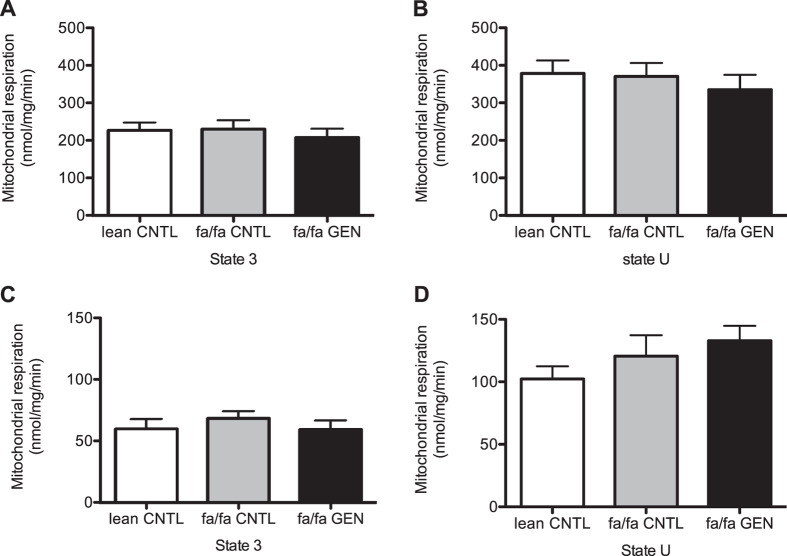
Oxidative capacity of isolated mitochondria from gastrocnemius muscle is not affected upon genistein consumption. (**A**) Coupled (ADP-driven) mitochondrial state 3 respiration upon pyruvate, (**B**) Uncoupled (FCCP-stimulated) state U respiration on pyruvate. (**C**) State 3 on palmitoyl-CoA and D) State U on palmitoyl-CoA in lean and fa/fa rats at 14 weeks of age.

Intrinsic mitochondrial respiratory capacity on either carbohydrate- or fatty acid-derived substrates was determined by measuring mitochondrial respiration upon either pyruvate or palmitoyl-CoA with carnitine. However, neither genistein treatment nor genotype significantly affected either maximal coupled (ADP-driven respiration, state 3) (P = 0.76) or uncoupled (FCCP-stimulated respiration, state U) (P = 0.68) mitochondrial respiration on pyruvate (Fig. 5A,B) or palmitoyl-CoA with carnitine (P = 0.62 and P = 0.29 for state 3 and state U respectively) (Fig. 5C,D). These data indicate that dietary genistein did neither affect mitochondrial density nor intrinsic mitochondrial function.

## Discussion

Genistein, a natural food compound mainly present in soybeans, has been shown to alleviate symptoms associated with T2DM. Nevertheless, the exact mechanism(s) underlying effects of genistein on glucose homeostasis under diabetic conditions remain poorly understood. In myotubes, genistein has been shown to improve glucose uptake^9^ and fatty acid handling^10^, implying improved oxidative capacity in muscle. In rodent models of T2DM, genistein has been reported to improve glucose homeostasis^11^,^12^,^13^,^14^ and attenuate ROS-generation^17^,^20^. Because skeletal muscle plays a central role in the maintenance of insulin sensitivity^29^, and muscle mitochondrial dysfunction is associated with insulin resistance and T2DM^21^,^22^, we here examined whether genistein influences muscle mitochondrial function and superoxide production in a rat model of progressive insulin resistance. We hypothesized that dietary supplementation of genistein inhibits the transition of the hyperinsulinemic normoglycemic state (pre-diabetes) towards full blown T2DM by improving skeletal muscle mitochondrial oxidative capacity. However, we found no evidence for genistein supplementation to improve whole-body glucose tolerance, skeletal muscle oxidative capacity, or ROS-induced stress during the maturation of ZDF rats. Therefore, our data argue against a beneficial role of genistein in the prevention of the development of advanced insulin resistance. In fact, increased fasting glucose levels in genistein-supplemented rats and elevated glucose levels at several time points after and oral glucose load may even be interpreted as unwarranted.

The ZDF rat is a well-known model of progressive insulin resistance^23^. As anticipated, we found that fa/fa rats had higher leptin levels, food intake, and body weight in comparison to lean rats. At 14 weeks of age fa/fa rats had significantly elevated fasting glucose levels and muscle fat content compared to lean rats, implying that fa/fa rats had developed an advanced state of insulin resistance indeed. In addition, no major differences were found in mitochondrial function upon either pyruvate or palmitoyl-CoA with carnitine between mature fa/fa and lean rats, which is consistent with findings in previous studies using the same rat model^24^,^30^. Hence, studying effects of genistein on glucose homeostasis and muscle oxidative capacity in fa/fa ZDF rats during maturation could provide more insight in the potential of genistein to inhibit the transition from a pre-diabetic state toward advanced stages of insulin resistance.

Interestingly, we found that after 8 weeks dietary intervention, fa/fa rats that consumed the genistein-enriched diet (fa/fa GEN) had similar body weights compared with the untreated fa/fa (fa/fa CNTL) rats. Also food intake and IMCL content were not different upon genistein. However, genistein had a negative rather than a positive effect on hyperglycemia as fasting glucose levels were slightly elevated in fa/fa GEN rats compared with the fa/fa CNTL rats. Also, blood glucose levels at 60 and 120 minutes after a glucose bolus were slightly higher in fa/fa GEN rats compared with fa/fa CNTL rats. Thus, unexpectedly, our data show a more unfavorable rather than favorable effect of dietary genistein on glucose homeostasis under conditions of insulin resistance. A previous study using nongenetic obese diabetic mice that were generated by high fat feeding and streptozotocin injections, also examined the effect of genistein on whole-body glucose homeostasis^15^. Whereas there were also no effects of genistein on body weight or fat deposition in these mice, hyperglycemia was blunted and glucose tolerance improved upon 0.025% dietary genistein intake^15^. Importantly, streptozotocin injections rendered these mice diabetic due to compromised pancreatic β-cell function. Thus, the mechanism underlying insulin resistance in streptozotocin diabetic mice is essentially different from the fa/fa ZDF model, where muscle insulin resistance appears to be the primary defect. The essential difference in the underlying mechanism may explain the discrepancy between the beneficial effects of genistein on glucose tolerance in streptozotocin treated mice vs. our observations in fa/fa rats. Additionally, the actions of genistein (and other isoflavones), appear to depend on the complex interaction of several factors, including the its duration and concentration of dosage regimens, but also age and sex of the individual have been reported to affect the action of genistein^31^.

Although genistein had no beneficial effects on whole-body glucose homeostasis, genistein could still have an effect on substrate metabolism at the level of skeletal muscle. Type 2 diabetes patients have been characterized by increased levels of skeletal muscle fat content^32^,^33^,^34^ accompanied by excessive ROS generation and oxidative stress^35^,^36^,^37^. As a consequence, enhanced lipid peroxidation leads to increased production of lipotoxic by-products that could negatively affect muscle substrate metabolism^38^,^39^, and subsequently, contribute to the development of T2DM^35^,^40^.

Since recent data point toward a beneficial role of genistein against ROS^17^,^18^,^19^,^20^, we examined the effect of genistein on mitochondrial superoxide production and oxidative damage in skeletal muscle under insulin resistance conditions. To the best of our knowledge, this is the first study demonstrating that genistein supplementation does not directly affect skeletal muscle mitochondrial superoxide anion radical formation as assessed by ESR. This was accompanied by lack of effect of genistein treatment on oxidative stress levels as was reflected by comparable levels of carbonylated proteins^41^. Analogous to our data on oxidative stress, UCP3 protein levels, another postulated protective mechanism against lipotoxicity^26^,^27^,^28^ suggested to be activated by genistein^10^, remain unaffected. It has been reported that effects of genistein on oxidative stress are less pronounced under insulin resistance conditions. In this context, in human umbilical vein endothelial cells, genistein had protective effects against ROS-induced apoptosis and inhibition of cell proliferation under normal glucose conditions (5 mM)^42^. Though, these protective effects were less effective at high glucose levels (25 mM) mimicking diabetic conditions^42^. These data along with our findings in the present study imply that genistein alone does not effectively lower oxidative stress in skeletal muscle under conditions of insulin resistance in ZDF rats.

Nevertheless, it is important to note that our focus was on mitochondrial ROS production and markers of ROS-induced damage in skeletal muscle. In livers of diet-induced insulin-resistant rats it has been reported that a dose of 1 mg/kg genistein via oral gavage did prevent oxidative damage^17^. Additionally, in a mouse model of focal cerebral ischemia injury, a genistein dose of half the concentration of what was used in the present study (2.5–10 mg/kg vs. 15–20 mg/kg per day respectively) could decrease ROS generation directly as was shown by reduced H_2_O_2_ levels^43^. Tissue-specific effects of genistein on oxidative stress could be induced due to the presence of tissue-specific estrogen receptor (ER)α/ERβ ratios^44^ and tissue differences may help to understand the aberrant differences between these studies and ours.

To elucidate the effect of genistein on skeletal muscle oxidative capacity in more detail, we measured mitochondrial density and function. Comparable OXPHOS levels between fa/fa CNTL and fa/fa GEN demonstrate that consuming a diet enriched with 0.02% (w/w) genistein during 8 weeks did not influence mitochondrial density in 14-week-old ZDF rats. Also, genistein supplementation did not improve intrinsic mitochondrial function. This finding was displayed by comparable levels of maximal coupled (ADP-driven) and uncoupled (FCCP-stimulated) mitochondrial respiration on either palmitoyl-CoA or pyruvate, in fa/fa CNTL and fa/fa GEN groups. Thus, genistein supplementation during the development of advanced stages of insulin resistance does not affect mitochondrial density, or influence intrinsic mitochondrial function. Our data on mitochondrial density and function match our finding that genistein did not reduce oxidative stress under diabetic conditions. Whereas others have shown, using the same model of progressive insulin resistance, that drug or diet interventions^45^,^46^ indeed can ameliorate or delay the progression of insulin resistance, our data indicate that genistein supplementation in a dose of 0.02% (w/w) does not alleviate the transition of the hyperinsulinemic normoglycemic state towards advanced insulin resistance by improving skeletal muscle mitochondrial oxidative capacity in ZDF rats. The dosing of the present study was based on previous animal work in which effects of genistein on other physiologically relevant parameters like fat oxidation, glucose and lipid regulating enzymes and PPAR activation parameters were reported^10^,^11^,^12^,^47^,^48^. Oral bioavailability of genistein in male rats is approximately 6.8%^49^. The half-life time of an orally provided 4 mg/kg bw dose of genistein was proven to be 16.8 hours^49^ with plasma values peaking after 0.5 hours^49^.

In the present study we mixed genistein with the diet. This resulted in consumption of 10–12 mg genistein per animal over a 24-hour period. As rats mainly eat throughout the dark phase, elevation of plasma levels of genistein are induced during the dark phase and with a half-life time of 16.8 hours, are likely to be significantly elevated relative to control values during the light phase. We did not aim to test a genistein dose-effect relationship. Hence, we cannot exclude that genistein, if provided in other dosages than tested, may affect insulin resistance or related parameters.

In conclusion, we here studied the effects of genistein on glucose homeostasis and skeletal muscle metabolism in a rat model of progressive insulin resistance and development of T2DM. Our data show that dietary supplementation of 0.02% (w/w) genistein had no beneficial effects on whole-body glucose tolerance, skeletal muscle oxidative stress, or intrinsic mitochondrial function in ZDF rats. Since skeletal muscle is an important site of insulin resistance and T2DM development, we suggest that dietary supplementation of pure genistein does not alleviate symptoms associated with T2DM progression. Moreover, we here showed that genistein feeding of hyperinsulinemic hyperglycemic ZDF rats does not prevent or inhibit the transition from a prediabetic state toward advanced insulin resistance and development of T2DM. In fact, we even demonstrated that under diabetic conditions, genistein could give unfavorable effects on glucose homeostasis.

## Materials and Methods

### Animals

Six-week old leptin receptor deficient ZDF rats (ZDF/Gmi, fa/fa, n = 12) and their homozygote littermates (ZDF/Gmi, +/+ (lean), n = 12) were purchased from Charles River (Maastricht, The Netherlands). Per genotype, the animals were housed in pairs in a controlled environment (21 °C) with a 12h light : 12h dark cycle. During the entire study, rats had *ad libitum* access to tap water and diet (Purina 5008; 16.7 energy percentage (En%) fat, 56.4 En% carbohydrates, and 26.8 En% protein, Altromin Germany). Reported studies demonstrated that addition of genistein to the diet at a dose of 0.02% (w/w) positively affects lipid metabolism, reduces oxidative stress, and has anti-diabetic potential^10^,^11^,^12^,^47^,^48^. Therefore, from 6 weeks of age and onward, rats were fed *ad libitum* the standard Purina diet either with or without 0.02% (w/w) genistein (Shanghai Dnd Pharm-technology Co. Inc.) for the duration of 8 weeks (n = 6 per group). Body weight and food consumption was monitored weekly throughout this 8-week period of dietary intervention. Intraperitoneal glucose tolerance tests (IPGTT) were performed at 13 weeks of age. At the age of 14 weeks, rats were sacrificed by cervical dislocation after a short sedation with a mixture of CO_2_ and O_2_ (4:1). Subsequently, gastrocnemius muscles were rapidly dissected and partly (~2 grams) directly used for the isolation of skeletal muscle mitochondria. Gastrocnemius muscle was also rapidly frozen for histology analyses and Western blotting assays in liquid N_2_-cooled isopentane and stored at −80 °C. All experiments were performed in accordance with the approved guidelines and regulations and complied with the principles of laboratory animal care. All protocols were approved by the Maastricht University animal ethics committee.

### Glucose tolerance tests and plasma measurements

For IPGTTs, rats were fasted for 4 hours and subsequently injected intraperitoneally with a glucose bolus (1 g/kg body weight). Blood glucose levels were measured in vena saphena blood samples prior to (t = 0) and at 5, 15, 30, 60, 90, and 120 minutes after the glucose administration using a glucose meter (LifeScan, Milpitas, CA, USA). Additionally, blood samples were collected in K-EDTA coated tubes, centrifuged at 1000 *g* for 10 minutes and plasma samples were frozen in liquid nitrogen and stored at −80 °C until analyses. Leptin levels were established with a rat/mouse ELISA kit from Millipore (Millipore, Billerica, MA, USA).

### Mitochondrial isolation

Within 30 seconds after sacrificing the animals, both gastrocnemius muscles were excised and processed on ice for mitochondrial isolation. In brief, the left gastrocnemius and the distal pieces of the right gastrocnemius muscle were immediately placed into ice-cold medium containing 100 mM sucrose, 50 mM KCl, 20 mM K^+^-TES, 1 mM EDTA and 0.2% (w/v) bovine serum albumin (BSA) (pH 7,4; with KOH) and processed for mitochondrial isolation according to Nabben *et al.*^50^. The protein concentration in the mitochondrial pellet was measured using fluorescamine (Fluram^®^, Fluka, Zwijndrecht, the Netherlands) with BSA as a standard^51^. Freshly isolated mitochondria were used immediately for electron spin resonance (ESR) spectroscopy and mitochondrial respirometry.

The medial part of the right gastrocnemius muscle was trimmed from visible fat and blood, embedded in Tissue-Tek (Sakura Finetek, Zoeterwoude, the Netherlands) and rapidly frozen in liquid nitrogen-cooled isopentane (2-methyl-butane, Fluka, Zwijndrecht, the Netherlands). Samples were stored at −80 °C until further analysis.

### Intramuscular lipid storage

To quantify the neutral lipid storage in skeletal muscle, cryosections (5 μm) of gastrocnemius muscles were stained with oil red O (ORO) as previously described^52^. ORO staining was combined with laminin staining (L-9393. Sigma, St Louis, USA) to visualize the cell membranes. Sections were examined using a Nikon E800 fluorescence microscope (Nikon, Amsterdam, The Netherlands). Digital images were captured and processed using Lucia G/F 5.49 image analysis software (Nikon, Dusseldorf, Germany). IMCL content was expressed per cell surface area and normalized to the lean control group.

### Reactive oxygen species (ROS) production

Mitochondrial ROS production was measured using electron spin resonance (ESR) spectroscopy, as described earlier^53^,^54^,^55^. Briefly, freshly isolated mitochondria (0.1 mg/ml protein) from gastrocnemius muscle were pre-incubated in mitochondrial respirometry medium (without malate), consisting of 100 mM sucrose, 50 mM KCl, 20 mM K^+^-TES (pH 7.2), 2 mM MgCl_2_, 1 mM EDTA, 4 mM KH_2_PO_4_, and 0.1% (w/v) bovine serum albumin (BSA), at 37 °C for 5 minutes. Subsequently, malate (3 mM) and pyruvate (5 mM) were added in the same concentrations as for the respirometry measurements. Charcoal purified 5,5-dimethyl-1-pyrroline N-oxide (DMPO) (100 mM; Sigma-Aldrich, St Louis, MO, USA) was added as a spintrap. Superoxide anion radical derived DMPO˙-OH signals were measured in triplicate (starting from the pre-incubation) on a Bruker EMX 1273 spectrometer^55^. Using the WIN-EPR spectrum quantification software (version 2.11, Bruker, Rheinstetten, Germany) spectra were quantified by summation of the peak heights from the four peaks of the DMPO˙-OH spectrum as previously described^26^. Values are expressed as percentage of the radical signal intensity of the lean control rats.

### Mitochondrial respirometry

Respiration rates were measured in freshly isolated mitochondria at 37 °C using a two-chambered Oxygraph (Oroboros Instruments, Innsbruck, Austria). Mitochondria (0.1 and 0.25 mg/ml mitochondrial protein for pyruvate and palmitoyl-CoA, respectively) were incubated in medium consisting of 100 mM sucrose, 50 mM KCl, 20 mM K^+^-TES (pH 7.2), 2 mM MgCl_2_, 1 mM EDTA, 4 mM KH_2_PO_4_, 3 mM malate and 0.1% (w/v) bovine serum albumin (BSA). Pyruvate (5 mM) was used as a carbohydrate-derived substrate and carnitine (2 mM) + palmitoyl-CoA (50 μM) as fatty acid-derived substrate. After addition of substrate, maximally coupled (state 3) respiration was measured by adding ADP (450 μM). Subsequently, basal (state 4) respiration was induced by the addition of the ATP-synthase inhibitor oligomycin (1 μg/ml). Maximal mitochondrial respiration capacity (state U respiration) was quantified by titrating the chemical uncoupler carbonyl cyanide p-trifluoromethoxyphenylhydrazone (FCCP). Mitochondrial respiration was expressed as nmol O_2_ per mg mitochondrial protein per minute.

### Western blot analysis

To obtain a marker for mitochondrial density we used an antibody that selectively recognizes structural components of the electron transport chain and oxidative phosphorylation (rodent OXPHOS antibody cocktail, MS604; MitoSciences, Eugene, OR, USA). As lipotoxicity may contribute to the development of insulin resistance^38^, especially under conditions of elevated production of reactive oxygen species^37^,^56^ and UCP3 has been postulated to modulate lipotoxicity^26^,^27^,^28^, we also examined UCP3 protein levels. Hence, western blotting was performed in homogenates of the right gastrocnemius muscle as described previously^24^,^51^. In short, standard SDS-PAGE blocking and incubation protocols were followed. Blots were incubated overnight at 4 °C with primary antibodies against electron transport chain complexes proteins (OXPHOS) or UCP3 (rabbit polyclonal antibody to UCP3; ab3477; Abcam, Cambridge, UK). After incubation with the primary antibodies, appropriate secondary antibodies (Rockland Immunochemicals, TeBu, Heerhugowaard, The Netherlands) were applied. Both OXPHOS and UCP3 blots were visualized and quantified using the Odyssey Near Infrared Imager (Licor Biosciences, Westburg, Leusden, The Netherlands).

In addition, as a marker for oxidative damage, the amount of protein carbonyls in isolated mitochondrial fractions was quantified using an Oxyblot oxidized protein detection kit (Millipore, Billerica, MA, USA) according to Lenaers *et al.*^24^. All protein contents were expressed as arbitrary units (AU).

### Statistics

Results are expressed as means ± SEM. For examining the effect of genistein, comparisons of data were made between lean CNTL, fa/fa CNTL, and fa/fa GEN groups through one-way ANOVA analyses followed by a Newman-Keuls posthoc test. To analyze the effect of genistein on glucose clearance during the IPGTT, mixed ANOVA analysis was performed with a Tukey posthoc test. Simple main effects were explored in order to determine the differences between groups at each time point. Per time point separate one-way ANOVAs were performed and multiple comparisons were obtained to establish the genistein effect per group at each time point. Statistical analyses were performed using GraphPad Prism 5.0a Macintosh Version. SPSS for Macinthosh 20.0 software was used for mixed ANOVA analysis. Statistical significance was set at P < 0.05.

## Additional Information

**How to cite this article**: van Bree, B. W. J. *et al.* A genistein-enriched diet neither improves skeletal muscle oxidative capacity nor prevents the transition towards advanced insulin resistance in ZDF rats. *Sci. Rep.* **6**, 22854; doi: 10.1038/srep22854 (2016).
